# The Impact of HIV and Malaria Coinfection: What Is Known and Suggested Venues for Further Study

**DOI:** 10.1155/2009/617954

**Published:** 2009-08-09

**Authors:** Sarah Hochman, Kami Kim

**Affiliations:** ^1^Department of Medicine, Division of Infectious Diseases, Montefiore Medical Center, Albert Einstein College of Medicine, Ullmann 1205, 1300 Morris Park Avenue, Bronx, NY 10461, USA; ^2^Department of Microbiology and Immunology, Albert Einstein College of Medicine, Ullmann 1225, 1300 Morris Park Avenue, Bronx, NY 10461, USA

## Abstract

HIV and malaria have similar global distributions. Annually, 500 million are infected and 1 million die because of malaria. 33 million have HIV and 2 million die from it each year. Minor effects of one infection on the disease course or outcome for the other would significantly impact public health because of the sheer number of people at risk for coinfection. While early population-based studies showed no difference in outcomes between HIV-positive and HIV-negative individuals with malaria, more recent work suggests that those with HIV have more frequent episodes of symptomatic malaria and that malaria increases HIV plasma viral load and decreases CD4+ T cells. HIV and malaria each interact with the host's immune system, resulting in a complex activation of immune cells, and subsequent dysregulated production of cytokines and antibodies. Further investigation of these interactions is needed to better define effects of coinfection.

## 1. Introduction

HIV and malaria have similar global distributions, with the majority of those affected living in sub-Saharan Africa, the Indian subcontinent, and Southeast Asia. Given the overlap of their geographic distribution and resultant rates of coinfection, interactions between the two diseases pose major public health problems. Together they accounted for over 3 million deaths in 2007 [1, 2], and millions more are adversely affected each year. Malaria and HIV/AIDS are both diseases of poverty and contribute to poverty by affecting young people who would otherwise enter the workforce and contribute to the local economy.

Malaria is caused by the protozoan parasite *Plasmodium* and is transmitted by *Anopheles* mosquitoes. It is endemic in most tropical and subtropical regions of the world. Of the four *Plasmodium* species that infect humans, *P. falciparum* is the most virulent and is responsible for the majority of morbidity and mortality due to malaria. Worldwide, 1.2 billion people are at risk for malaria infection, resulting in 500 million infections and more than 1 million deaths each year. The majority of these deaths occur in young children in sub-Saharan Africa, where one in every five childhood deaths is due to malaria [1]. Aside from young children, pregnant women are also heavily affected [3], with resultant effects on maternal health and birth outcomes. While recent data indicates the number of malaria infections per year is decreasing (247 million malaria cases in 2006) the number of deaths attributable to malaria remains unchanged [4].

Areas of the world with high rates of malaria also carry a heavy burden of HIV. There are 33 million people living with HIV worldwide, with 22.5 million in sub-Saharan Africa alone. This results in an estimated overall prevalence of 5% in sub-Saharan Africa, with some countries reporting prevalence rates of greater than 25%. While new HIV infections in adults and children have decreased since 2005, there were an estimated 2.5 million children living with HIV in 2007, nearly 90% of whom are in sub-Saharan Africa. It is estimated that 2.1 million deaths in 2007 were due to HIV, of which 1.6 million occurred in sub-Saharan Africa, making HIV/AIDS the number one cause of mortality in that region [2].

## 2. Physiologic Impact of Malaria

*Falciparum* malaria has a spectrum of clinical presentations, ranging from asymptomatic parasitemia in patients with immunity to severe anemia, cerebral malaria, multiorgan failure, or death.

Anemia is most frequently seen in young children and pregnant women [5] and can be seen in acute infection as well as with chronic repeated malarial infections. The underlying causes of severe malarial anemia are likely multifactorial. Extravascular and/or intravascular hemolysis of both infected and uninfected erythrocytes plays a role: changes in surface proteins on infected erythrocytes lead to increased clearance of these cells [6], while noninfected red blood cells are destroyed in the spleen during acute infection [7]. This leads to hemolysis and depletion of iron stores. Bone marrow suppression also plays an important role in the pathogenesis of malarial anemia. The normal response to hemolytic anemia is enhanced secretion of erythropoietin, leading to stimulation of erythropoiesis, but this mechanism seems to be defective in patients with malaria. During acute infection, abnormalities are seen in erythroid progenitors [8], while dyserythropoiesis (abnormal production of red cells) is observed in chronic infection [9].

Cerebral malaria and other end-organ damage is mediated through interactions between infected red blood cells and host receptors on the blood vessel wall, resulting in adherence and sequestration of infected red blood cells in the postcapillary venules, obstruction of blood flow, and subsequent tissue damage [10]. Patients who survive cerebral malaria may suffer from long-term mental and psychological deficits [11]. Renal complications are common and may present as acute renal failure due to the effects of sequestrated infected red blood cells or with nephrotic syndrome, due to deposition of antigen-antibody complexes within glomeruli. *P. falciparum* can also result in severe anemia, low birth weight, and maternal death during pregnancy [3, 12].

## 3. Physiologic Impact of HIV

HIV infects and depletes CD4+ T lymphocytes, putting patients at risk for opportunistic infection and malignancy, the major causes of death due to HIV and AIDS. However, it also has effects on the systemic inflammatory response, causing activation and/or apoptosis in a variety of immune cells as well as elevated levels of proinflammatory cytokines and chemokines in plasma and lymph nodes. This immune activation, rather than being a reflection of antiviral immunity, is associated with HIV-1 disease progression [13]. It is also a potential means by which HIV affects disease course and outcome in other infections, such as malaria.

## 4. Impact of HIV and Malaria Coinfection

While no population-based studies are available to determine the number of patients affected by coinfection and the impact of coinfection on a population, a mathematical model applied to previously published data was used to estimate the impact of HIV-1 on malaria in sub-Saharan Africa. It estimated that an additional 3 million cases of malaria and 65 000 additional malaria-related deaths annually are due to the impact of HIV [14]. Rates were highest in countries with high HIV prevalence and unstable malaria transmission.

Cohort studies have shown that malaria infection causes an increase in plasma HIV viral load, even during asymptomatic parasitemia. HIV viral load returned to baseline eight weeks after acute malaria infection [15]. It has also been demonstrated that CD4+ T lymphocytes decline temporarily during clinical malaria episodes in HIV-infected and HIV-uninfected patients [16] and that repeated malaria infections are associated with a more rapid decline in CD4+ T lymphocytes over time [17], suggesting that malaria may lead to faster disease progression from HIV to AIDS.

While early studies found no association between HIV and malaria disease severity in either adults or children [18, 19] more recent research has shown that HIV infection predisposes to more frequent episodes of symptomatic malaria [20] and more episodes of severe or complicated malaria including death in both children and adults [21–24], see Table 1. An inverse relationship was found between incidence of severe malaria and CD4+ T lymphocyte counts. Generally, patients with HIV respond to standard malaria regimens. While infection with HIV has been associated with an increased rate of malaria treatment failure, this was due to re-infection with new malaria strains, rather than recrudescence of prior infection [20].

In HIV-uninfected women, risk for symptomatic or placental malaria decreases with each subsequent pregnancy. In HIV-infected women this gravidity-specific pattern is altered, such that multigravidae women carry the same risk of disease as primigravidae women [25]. Pregnancy-associated malaria is associated with increased risk of maternal anemia, intrauterine growth restriction, and delivery of preterm, and low-birth-weight infants [26]. Given the lack of gravidity-specific protection against malaria seen with HIV infection in pregnant women, HIV puts more pregnancies at risk for complications associated with malaria.

In a cohort study in Kenya, HIV-coinfected women had higher placental parasite densities and higher rates of antenatal malaria transmission than did HIV-uninfected women [26]. Maternal antibody to variant surface antigens (VSAs) on malaria-infected erythrocytes plays an important role in pregnancy-related immunity to malaria. Sera from HIV-infected mothers when analyzed by flow cytometry contained fewer antibodies to VSAs in both placental and pediatric isolates of malaria than did sera from HIV-uninfected mothers [27]. Additionally, assays using plasma or purified IgG from HIV-infected or HIV-uninfected primi- or multigravidae women found that HIV-uninfected multigravidae women had high levels of opsonic phagocytosis of infected erythrocytes, which was due to IgG1 and IgG3 specific for VSA. Opsonic phagocytosis was not seen with plasma or purified IgG from HIV-uninfected primigravidae women or HIV-uninfected men. HIV-infected multigravidae women had significantly lower plasma opsonizing activity than did their HIV-uninfected counterparts [28].

Cohort studies in Cameroon show that malaria infection during pregnancy may increase the risk of mother-to-child transmission of HIV [29, 30]. One potential mechanism for this was evaluated in vitro, where binding of recombinant *P. falciparum* adhesin to chondroitin sulfate A on human placental cells increased HIV-1 replication in those cells, possibly via TNF-alpha stimulation [31]. 

## 5. Immune Response to Malaria

Proinflammatory (Th1) cytokines such as TNF-alpha are thought to play an important role in malaria pathogenesis and in cerebral malaria in particular, as they increase surface expression of adhesion molecules on endothelial cells, promoting parasite attachment. Activated CD4+ T lymphocytes stimulate macrophages to produce TNF-alpha, which leads to cerebral malaria in mouse models [32].

In humans, clusters of cytokines may help discriminate between mild, severe, and cerebral malaria. In an adult population in a malaria-endemic region of India, high levels of IL-12, IL-5, and IL-6 discriminated severe forms of malaria from mild malaria. Levels of IL-1beta, IL-12, and IFN-gamma helped to discriminate cerebral malaria from severe malaria, with high IL-1beta levels being associated with cerebral malaria, and high IL-12 and IFN-gamma levels being associated with severe malaria [33]. In a pediatric population in Mali, high levels of IL-6 and IL-10 helped to discriminate both severe malaria from mild malaria and cerebral malaria from severe malaria, whereas IL-1beta and IL-12 did not differ significantly among groups [34]. While there are many studies that associate patterns of cytokines to disease, results may be different depending on the cohort population. Thus, there is an association between elevations in certain cytokines and disease outcomes, but it is hard to generalize these associations to different patient populations.

Adaptive immunity to malaria is thought to confer protection against febrile parasitemia but does not prevent parasitemia. This is due in part to the development of antibodies against proteins on the infected erythrocyte surface, notably VSA [35].

The effect of malaria on dendritic cells, key players in both the early stages of adaptive immunity and innate immunity, is not clear. Urban et al. found that *P. falciparum*-infected erythrocytes adhere to human dendritic cells, inhibit their maturation, and subsequently reduce their capacity to stimulate T cells [36]. However, mouse studies using *P. chabaudi* found that infected erythrocytes induce maturation of dendritic cells, stimulate IL-12 and IFN-gamma production, and cause CD4+ T lymphocyte proliferation [37]. While these two studies reported conflicting findings, one unifying explanation for both was seen in another mouse model, using *P. yoelii*. Overstimulation of dendritic cells by toll-like receptors makes them refractory to further activation in *P. yoelii*-infected mice [38]. As malaria infection progresses and parasitemia increases exponentially, dendritic cells will develop toll-like receptor resistance. This would result in dendritic cell dysfunction in later stages of infection, as was seen by Urban et al., while the findings of Ing et al. may be more consistent with the early stages of malaria.

## 6. Immune Response to HIV

Proinflammatory cytokines play an important role both in control and pathogenesis of HIV infection. During infection, viral particles are taken up by antigen presenting cells (APCs), which are then recognized by CD4+ T lymphocytes, causing activation and release of IL-2 and IFN-gamma. These proinflammatory cytokines in turn stimulate CD8+ T lymphocytes, which control viremia. It is not known if this inflammatory response seen in HIV has an effect on the adherence and sequestration seen in malaria. However, HIV upregulates adhesion molecules on endothelial cells, which may compound the adherence and sequestration seen in malaria [39].

HIV also dysregulates pathways of cytokine expression, such that production of the proinflammatory cytokines IL-12 and IFN-gamma is decreased and expression of the anti-inflammatory cytokine IL-10 is increased [40]. As HIV progresses clinically to AIDS there are effects on innate immunity, with progressive loss of T lymphocyte responses to common recall antigens [41]. Increased IL-10 has been shown to play a role in this impaired innate immune response in AIDS patients [42].

The impaired innate immune response in patients with AIDS may in part account for the increased rates of symptomatic malaria seen in cohort studies. However, the decreased production of IL-12 and IFN-gamma seen in HIV is confounding, as high levels of these proinflammatory cytokines are associated with severe malaria in clinical studies [33], and previously mentioned cohort studies have found higher rates of severe or symptomatic malaria in subjects with HIV.

Increased expression of IL-10 also appears to play a role in loss of adaptive immunity. IL-10 impairs T helper type 1 (Th1) responses [42]. Dendritic cells In HIV/AIDS are functionally impaired, producing less IL-12 and more IL-10, disrupting the IL-12/IFN-gamma signaling pathway and contributing to problems with adaptive immunity [40]. It is not clear if this impairment is due to direct HIV infection of dendritic cells, or that indirect effects of chronic antigenic stimulation or exposure to virally induced proteins cause dendritic cell dysfunction. Given the important role of dendritic cells in adaptive immunity to malaria, the effects of HIV on dendritic cell dysfunction may also contribute to the higher frequency of symptomatic parasitemia seen in cohort studies.

## 7. Effects of Malaria on the Endothelium and Blood-Brain Barrier

Cerebral malaria is a major cause of death due to *P. falciparum* in children under the age of five. Characterized by coma and/or seizures, it is associated with sequestration of parasitized red blood cells in the brain microvasculature. Occasionally brain edema and elevated intracranial pressure are seen.

Postmortem samples from children who died from cerebral malaria show activation of endothelial cells (with upregulation of ICAM-1) and macrophages (with increased macrophage scavenger receptor and sialoadhesin), and disruption of endothelial intercellular junctions (ZO-1, occludin, vinculin) in vessels containing sequestered parasitized red blood cells. No leakage of plasma proteins (fibrinogen, C5b-9, IgG) into brain parenchyma was seen, suggesting that the blood-brain barrier remains intact. However, there were elevations in cerebrospinal fluid albumin taken prior to death, which may indicate blood-brain barrier permeability [43].

In vitro studies have backed up some of these findings. Gillrie et al. showed that parasite sonicates but not intact malaria-infected red blood cells disrupt the endothelial (dermal and pulmonary, not brain) barrier, revealed by discontinuous immunofluorescent staining of endothelial junction proteins, formation of interendothelial gaps in monolayers, and loss in total protein content of claudin 5 and redistribution of ZO-1 [44].

Additionally it appears that severity of malaria infection is associated with differential expression of tight junction proteins. Quantitative PCR of human umbilical vascular endothelial cells (HUVECs) cultured with *P. falciparum* samples from patients with uncomplicated malaria showed increased mRNA levels of occludin, vinculin, and ZO-1. Those cultured with samples from severe malaria had no change in mRNA levels, and HUVECs cultured with *P. falciparum* from patients with cerebral malaria had decreased mRNA levels of occludin, vinculin, and ZO-1 [45]. This data suggests that infected erythrocytes can alter the expression of tight junction proteins in endothelial cells at the site of sequestration, influencing disease severity.

Not only are endothelial junction proteins affected, but also endothelial cells themselves seem to be influenced by infected erythrocytes, producing inflammatory mediators in response to malaria infection. Endothelial cells produce tissue factor when cultured with *P. falciparum*, and brain endothelial cells from patients dying of cerebral malaria and from those with malaria who died of other causes also showed increased levels of tissue factor [46]. Tissue factor plays a role in coagulation via thrombin formation, and plays a role in inflammation, as it is upregulated by proinflammatory cytokines such as TNF-alpha and also induces expression of such cytokines [47].

Endothelial cells also upregulate adhesion molecules in the setting of *falciparum* malaria. In *P. falciparum* isolates from patients with complicated malaria, binding of infected red blood cells to human lung microvascular endothelial cells was observed and was primarily mediated through ICAM-1 and chondroitin sulfate (CSA) [48]. Mouse models of cerebral malaria have supported the role of ICAM-1. ICAM-1-deficient C57BL/6 mice infected with *P. berghei* ANKA were protected from mortality compared with C57BL/6 controls [49]. Additionally, lack of TNF receptor 2 conferred resistance to cerebral malaria in mice, thought to be due to blocking the upregulatory effects of TNF on ICAM-1 expression in brain microvascular endothelial cells [50].

Another adhesion molecule that is likely involved in cerebral malaria pathogenesis is CD36, a receptor with a wide tissue distribution that is also found on endothelial cells. Monoclonal antibodies to CD36 and ICAM-1 partially inhibited the binding of *P. falciparum*-infected red blood cells to human brain endothelium, suggesting that both are important for cytoadherence [51]. The rodent malaria strain *P. chabaudi* has been used to study malaria sequestration in mice, and *P. chabaudi* AS-infected red blood cells adhered to purified CD36 in vitro [52]. This is of particular interest when considering the role of HIV coinfection in malarial sequestration and effects on the blood-brain barrier, as CD36 expression on circulating monocytes is significantly higher in HIV-1 infected patients compared with healthy controls [53].

## 8. Effects of HIV on the Endothelium and Blood-Brain Barrier

Unlike malaria, HIV is known to invade and infect the brain parenchyma, causing HIV-associated encephalitis and/or HIV-associated dementia. HIV may infect astrocytes at low levels, and it can activate endothelial cells, but it does not infect endothelial cells. HIV is thought to enter brain parenchyma through the regulated transmigration of HIV-infected mononuclear cells across the blood-brain barrier, mediated by surface receptors on endothelial cells such as ICAM-1. Once across the barrier, HIV-infected cells recruit microglia and astrocytes, allowing for subsequent infection of these cells and spread of HIV within the CNS.

As HIV replicates within the CNS, it produces inflammatory mediators, including the chemokine CCL2/MCP-1 [54]. This chemokine has been shown to enhance blood-brain barrier permeability, with increased transmigration of HIV-infected monocytes and a loss of tight junction proteins [55]. In addition to CCL2/MCP-1, cytokines including TNF-alpha, IFN-gamma, IL-1beta, and IL-6 are increased in brain tissue and CSF of people with NeuroAIDS [54].

## 9. Drug Effects

Aspartic proteases play key roles in HIV and are thus important therapeutic drug targets. Aspartic proteases are also important in malaria parasites (as plasmepsins), and antiretroviral protease inhibitors seem to have both direct effects on *Plasmodium* and indirect effects related to cytoadherence and phagocytosis.

The antiretroviral protease inhibitors saquinavir, ritonavir, indinavir, nelfinavir, amprenavir, lopinavir, and atazanavir directly inhibit erythrocytic stages of *P. falciparum* grown in vitro at concentrations achieved in vivo [56, 57], and mice infected with *P. chabaudi* AS had a delay in patency and attenuation of parasitemia when given oral ritonanvir/saquinavir or ritonavir/lopinavir [58]. Some antiretrovirals also seem to exert an effect on the pre-erythrocytic stages of malaria. Using *P. berghei*, a rodent strain of malaria, saquinavir and lopinavir inhibited development of extra-erythrocytic liver stages in vitro. In vivo mouse studies using the rodent strain *P. yoelii* showed a reduction in liver parasite burden when lopinavir/ritonavir was administered [59].

The antiretroviral protease inhibitors ritonavir and saquinavir affect CD36-mediated cytoadherence, thought to play a role in cerebral malaria and other end-organ damage in severe malaria. The drugs decrease CD36 surface concentrations on C32 epithelial cells in culture, which was associated with a decrease in cytoadherence of parasitized erythrocytes [60]. No effect on ICAM-1 expression on cells was seen. In addition, these protease inhibitors affect nonopsonic phagocytosis of parasitized erythrocytes. Human macrophages exposed to ritonavir or saquinavir also had reduced surface values of CD36, with an associated decrease in nonopsonic phagocytosis. The NNRTI nevirapine had no effect on CD36 concentrations on either C32 cells or macrophages and did not affect cytoadherence or phagocytosis.

The antimalarial drug chloroquine has effects on HIV, inhibiting the production of infectious viral particles by impairing virus glycosylation. Chloroquine also has synergistic effects on HIV suppression with the protease inhibitors indinavir, ritonavir, and saquinavir at concentrations achieved with prophylaxis dosing [61]. In vitro studies have also shown a synergistic effect on malaria growth between the protease inhibitors ritonavir and saquinavir and both chloroquine and mefloquine [62].

An additional drug-related interaction between malaria and HIV involves the use of trimethoprim-sulfamethoxazole prophylaxis in HIV-infected patients. In rural Uganda, HIV-infected participants given trimethoprim-sulfamethoxazole had a 76% decrease in rates of malaria compared with when they were not receiving trimethoprim-sulfamethoxazole, and participants who received antiretroviral therapy plus trimethoprim-sulfamethoxazole had a 92% decrease in malaria rates [63]. It is important to note that the antiretroviral regimen used did not include a protease inhibitor.

## 10. Conclusions

Malaria and HIV affect millions of people across overlapping geographic distributions, and risk of transmission of both malaria and HIV may be increased due to coinfection. It has been observed that HIV-infected people in areas of malaria transmission have more frequent episodes of symptomatic parasitemia [20] and higher parasitemias than those without HIV [64]. Thus, there is likely a higher risk for increased transmission of malaria in these areas. Additionally, HIV-infected people have an increase in viremia during episodes of parasitemia [15, 65], leading to a potential increase in risk of HIV transmission. Given the sheer numbers of people living with HIV in sub-Saharan Africa, an area where malaria transmission is common, there is concern for a significant public health threat.

While some interactions between malaria and HIV are known, these interactions have not been extensively studied from an epidemiological perspective. Many aspects of the relationship between malaria and HIV remain unanswered. While malaria is a major cause of morbidity and mortality in children in sub-Saharan Africa [1], little is known regarding the contribution of HIV to rates of severe malaria, cerebral malaria, and malaria deaths in these children. Additionally, the findings of increased rates of symptomatic malaria, higher parasite densities, and poorer responses to malaria treatment in adults have not been studied in children.

While it is known that pregnant women with HIV have higher rates of symptomatic malaria [25], placental malaria, and malaria transmission to their children [26], it is not known if mother-to-child transmission of HIV is affected by malaria infection. And while it has been shown that malaria infection increases HIV viral load [15], it has yet to be demonstrated if this translates to higher rates of HIV transmission among populations. Additionally, it is not known if HIV increases rates of malaria transmission, even though it has been shown that HIV increases parasite density during malaria infection.

It is also unclear if HIV and malaria coinfection have an impact on treatment or clinical outcomes. Does HIV have an effect on resistance to antimalarial drugs, and are there more effective drugs to treat malaria in those who are also infected with HIV? Are there interactions between antiretrovirals used for HIV and antimalarial drugs currently in use, such as artemesin-derivatives? Also important to study are the effects of malaria on HIV prognosis: do the increases in viral load and decreases in CD4+ T lymphocytes seen during acute malaria infection have long term consequences on HIV progression to AIDS? These questions deserve investigative attention.

In addition to the need for population-based studies of coinfection, microbe-microbe interactions deserve further research efforts as well. It is likely that HIV and *P. falciparum* have a synergistic relationship, as both provoke an inflammatory response from immune cells, with subsequent effects on endothelial activation and blood-brain barrier permeability. Investigative methods that adequately mimic these pertinent clinical scenarios are needed. Thus, efforts to coculture malaria and HIV, and to observe the effects of each microbe during coculture, should be pursued. Examining the interactions between malaria and HIV in the setting of immune cells, endothelial models, and models of the blood-brain barrier would shed light on the effects on immune dysregulation, endothelial activation, and blood-brain barrier permeability.

Effective animal models of coinfection are also needed to look at systemic inflammatory responses and effects on end-organ damage, especially pertaining to cerebral malaria. While no animal model can fully mimic the effects of HIV and malaria coinfection in humans, it is impractical to invasively study dual infection in humans in a controlled, monitored setting. Established murine models of cerebral malaria rely on altered endothelial cell function and the immune response, both of which play a pivotal role in human disease [66]. A pertinent murine model of HIV infection and end-organ damage is the transgenic HIV_JR-CSF_ model, which has been used to study blood-brain barrier integrity [67]. Integrating these two models would allow for the development of an effective mouse model for HIV and malaria coinfection. Pursuing this development will allow researchers to identify clinical findings, such as outcomes in cerebral malaria, which have yet to be elucidated in population-based studies.

As mentioned previously, 500 million cases of malaria occur each year, causing 1 million deaths [1]. Most of these cases occur in sub-Saharan Africa, where 22.5 million people are living with HIV. Thus, millions of people are likely coinfected with HIV and malaria. Investigating the effects of coinfection through in vitro coculture models, animal models, and through clinical studies focused on the pertinent epidemiological and clinical issues outlined above is needed to better understand the impact of HIV and malaria coinfection on public health.

## Figures and Tables

**Table 1:** Effects of coinfection with HIV and malaria.

Interactions			
Type of Interaction	Pregnant women	Children	Adult men and nonpregnant women
Effect of HIV on malaria			
* *• ↑ Risk of infection	+	**?**	+
* *• ↑ Parasite density	+	**?**	+

Effect of malaria on HIV			
* *• ↑ Viral load	+	**?**	+
* *• ↑ Transmission	**?**	+	**?**
	(Data on vertical transmission to the fetus is contradictory)	(Through transfusion of unscreened blood for anemia)	(No definitive data, although ↑ viral load has previously been shown to correlate with ↑ sexual transmission)

Effects of dual infection			
* *• ↑ Severity of illness	+	+	+
* *• ↑ Frequency and severity of anemia	+	+	+
* *• ↑ Frequency of low birth weight	+	N/A	N/A
